# NOTCH3 attenuates cytotoxicity via RBPJ-dependent PVR upregulation to influence immunotherapy outcomes in colorectal cancer

**DOI:** 10.3389/fimmu.2026.1741261

**Published:** 2026-02-23

**Authors:** Qi Ma, Shuning Xu, Ning Ma, Ke Li, Ying Liu, Fei Ma

**Affiliations:** 1 Department of General Surgery, The Affiliated Cancer Hospital of Zhengzhou University, Henan Cancer Hospital, Zhengzhou, Henan, China; 2 Department of Medical Oncology, The Affiliated Cancer Hospital of Zhengzhou University, Henan Cancer Hospital, Zhengzhou, Henan, China; 3 Department of Oncology, Henan Provincial People’s Hospital, People’s Hospital of Zhengzhou University, Zhengzhou, Henan, China

**Keywords:** CRC, immunotherapy, mutation, notch3, PVR, RBPJ

## Abstract

**Introduction:**

This study aimed to elucidate the function of NOTCH3 in pan-cancer and CRC progression, its impact on the tumor immune microenvironment, and its value as a therapeutic target and predictive biomarker.

**Methods:**

We performed a multi-omics analysis of NOTCH3 alterations (expression, mutation, copy number variation, methylation) using data from The Cancer Genome Atlas (TCGA). Immune cell infiltration was assessed using multiple algorithms and single-cell RNA sequencing (scRNA-seq) data from CRC patients. In vitro functional experiments, including co-immunoprecipitation, chromatin immunoprecipitation (ChIP), luciferase reporter assays, and CD8^+^ T cell cytotoxicity co-cultures, were conducted in CRC cell line. An immune-competent mouse xenograft model was used to evaluate the efficacy of anti-NOTCH3 in combination with anti-PD-L1 therapy. Clinical validation was performed using independent immunotherapy-treated cohorts from the MSKCC database and our institutional cohort (102 patients) via immunohistochemistry and survival analysis.

**Results:**

NOTCH3 is frequently altered across multiple cancers. In CRC, high NOTCH3 expression correlated with poor survival and fostered an immunosuppressive microenvironment. Mechanistically, NOTCH3 transcriptionally upregulates the immune checkpoint molecule PVR by binding to the transcription factor RBPJ; this process is abrogated by NOTCH3 mutations (e.g., R1669H). NOTCH3-mediated PVR upregulation suppressed CD8+ T cell cytotoxicity. scRNA-seq analysis revealed enhanced PVR-TIGIT interactions between cancer and immune cells in NOTCH3-high tumors. In vivo, NOTCH3 depletion synergized with anti-PD-L1 therapy to inhibit tumor growth and increase CD8+ T cell infiltration. Clinically, NOTCH3 mutation or low expression independently predicted improved survival in immunotherapy-treated CRC and pan-cancer cohorts.

**Conclusion:**

NOTCH3 is a pivotal regulator of immune evasion in CRC via the RBPJ-PVR axis.

## Introduction

Colorectal cancer (CRC) is one of the most prevalent and lethal malignancies worldwide. In 2022, an estimated 1,926,118 new cases and 903,859 deaths were reported, accounting for 9.6% and 9.3% of all cancer cases and deaths, respectively (1). Globally, it ranks as the third most commonly diagnosed cancer and the second leading cause of cancer-related mortality. A high proportion of patients are diagnosed at an advanced stage, with approximately 45% presenting with distant metastasis at the time of diagnosis (2).

Targeted therapy, including immunotherapy, has gained significant attention over the past decade due to its relatively favorable objective response rate (3–5). For example, a recent phase II clinical trial involving 115 mismatch repair-deficient (dMMR) patients reported that the pathological response rate reached 98%, with a complete pathological response rate of 95% (5). In contrast, the response rate for microsatellite-stable/mismatch repair-proficient (MSS/pMMR) patients remains unsatisfactory (6),with an objective response rate (ORR) ranging from 27% to 35% (7, 8). However, dMMR accounts for less than 5% of all CRC cases (9), meaning that the pMMR CRC individual number that potentially benefit from immunotherapy is over six times larger than that of dMMR patients. Therefore, identifying biomarkers and potential therapeutic targets for CRC is critical for current research.

NOTCH3 (Neurogenic Locus Notch Homolog Protein 3) is the third discovered member of the NOTCH family, encoding a protein that interacts with its ligands to initiate NOTCH signaling pathway. The role of NOTCH3 has been widely reported in various biological processes, including carcinogenesis (10, 11) and drug resistance (12, 13). Alterations in NOTCH3 have also been observed in cancers (14). In bladder cancer, NOTCH3 directly interacts with SPP1, stimulating the PI3K/AKT signaling pathway (15). In CRC, NOTCH3 has been shown to promote tumor progression, while its disruption attenuates metastasis (16). Moreover, high NOTCH3 expression is significantly associated with macrophage infiltration and myeloid-derived suppressor cell (MDSC) recruitment, contributing to an immunosuppressive tumor microenvironment (17). Single-cell sequencing studies reveal that NOTCH family genes are essential for pericytes and cancer-associated fibroblasts (CAFs). NOTCH3 depletion leads to a reduced invasive niche (18). However, the detailed mechanisms linking NOTCH3 to the tumor microenvironment remain unclear; the impact of NOTCH3 alterations in CRC—particularly their influence on targeted therapy (e.g., immunotherapy)—has not yet been investigated.

In this study, we conducted a comprehensive analysis of NOTCH3 alterations across multiple biological dimensions using multi-omics data from TCGA. We systematically evaluated the association between NOTCH3 expression and clinical indicators, investigated its impact on immune infiltration patterns, and identified potential drugs associated NOTCH3. Furthermore, we elucidated the mechanistic role of NOTCH3 in modulating immune infiltration and immunotherapy response. Our findings not only advance the understanding of NOTCH3 in colorectal cancer but also identify it as a novel potential therapeutic target for CRC treatment.

## Results

### NOTCH3 alterations across cancers and CRC

**Figure 1:**
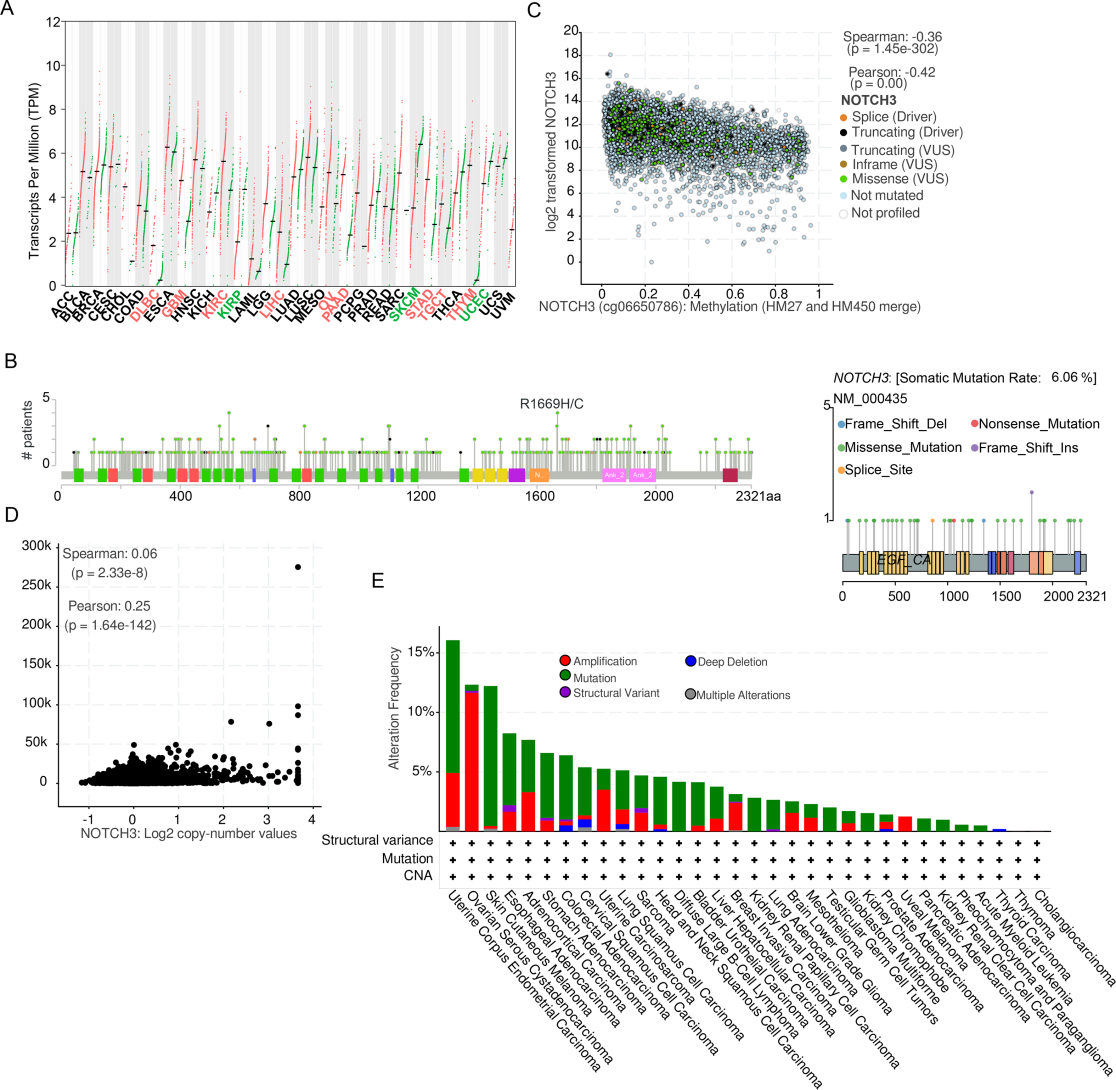
NOTCH3 alterations across human cancers and CRC. **(a)** NOTCH3 mRNA expression levels across TCGA cancer types green represents normal and red is cancer. The red labels indicate up-regulation in corresponding tumor type and the green labels mean down-regulation (statistically significant). **(b)** Distribution of NOTCH3 somatic mutations (lollipop plot showing mutation frequency and type across protein domains); left, pan-cancer; right, CRC (N = 534). **(c)** Negative correlation between NOTCH3 expression and cg06650786 methylation (scatter plot with Spearman R=-0.42, p=1.45e-302, N = 10967). **(d)** NOTCH3 expression stratified by copy number variation (N = 526). The x-axis is copy number of NOTCH3 across CRC samples and y-axis is NOTCH3 expression. **(e)** Combined alteration frequency (mutations + CNVs) across cancer (bar plot with CRC ranked 7th at 7%) patients (N = 10967).

We systematically analyzed NOTCH3 alterations across multiple cancer types from various molecular perspectives. At the transcriptional level, NOTCH3 expression exhibited a heterogeneous pattern among different cancers (**Figure 1a**) compared to their corresponding normal tissues. Elevated NOTCH3 expression was observed in glioblastoma (GBM), ovarian cancer (OV), pancreatic cancer (PAAD), and gastric cancer (STAD), Diffuse Large B-cell Lymphoma (DLBC), Kidney Renal Clear Cell Carcinoma (KIRC), Liver Hepatocellular Carcinoma (LIHC), Testicular Germ Cell Tumors (TGCT), Thymoma (THYM), while reduced expression was detected in melanoma (SKCM), kidney renal papillary cell carcinoma (KIRP), and uterine cancer (UCEC). In CRC, NOTCH3 expression was not significantly different. Mutation analysis revealed that NOTCH3 mutations were distributed sparsely across the coding region (**Figure 1b**, left), a pattern often associated with passenger mutations. This trend was also consistent in COAD (CRC, **Figure 1b**, right). The relationship between DNA CpG methylation (cg06650786) within NOTCH3 and its expression was analyzed, and a significant negative correlation was detected (**Figure 1c**, p =1.5e-302, Spearman correlation, R=-0.36). Additionally, NOTCH3 expression was also significantly correlated with copy number variations (CNVs, **Figure 1d**). Integrating CNV and mutational data, we found that UCEC exhibited the highest NOTCH3 alteration frequency (16%, **Figure 1e**), followed by ovarian cancer, melanoma, and esophageal cancer. In CRC, 7% samples harbored NOTCH3 alterations (mutations and/or CNVs), ranking 7th among all cancer types. In summary, our findings demonstrate that NOTCH3 alterations are prevalent across multiple cancers, with its expression significantly associated with both DNA methylation and copy number variations, suggesting that they may regulate the expression of NOTCH3.

### Higher NOTCH3 expression predicts poor survival in across cancers

**Figure 2:**
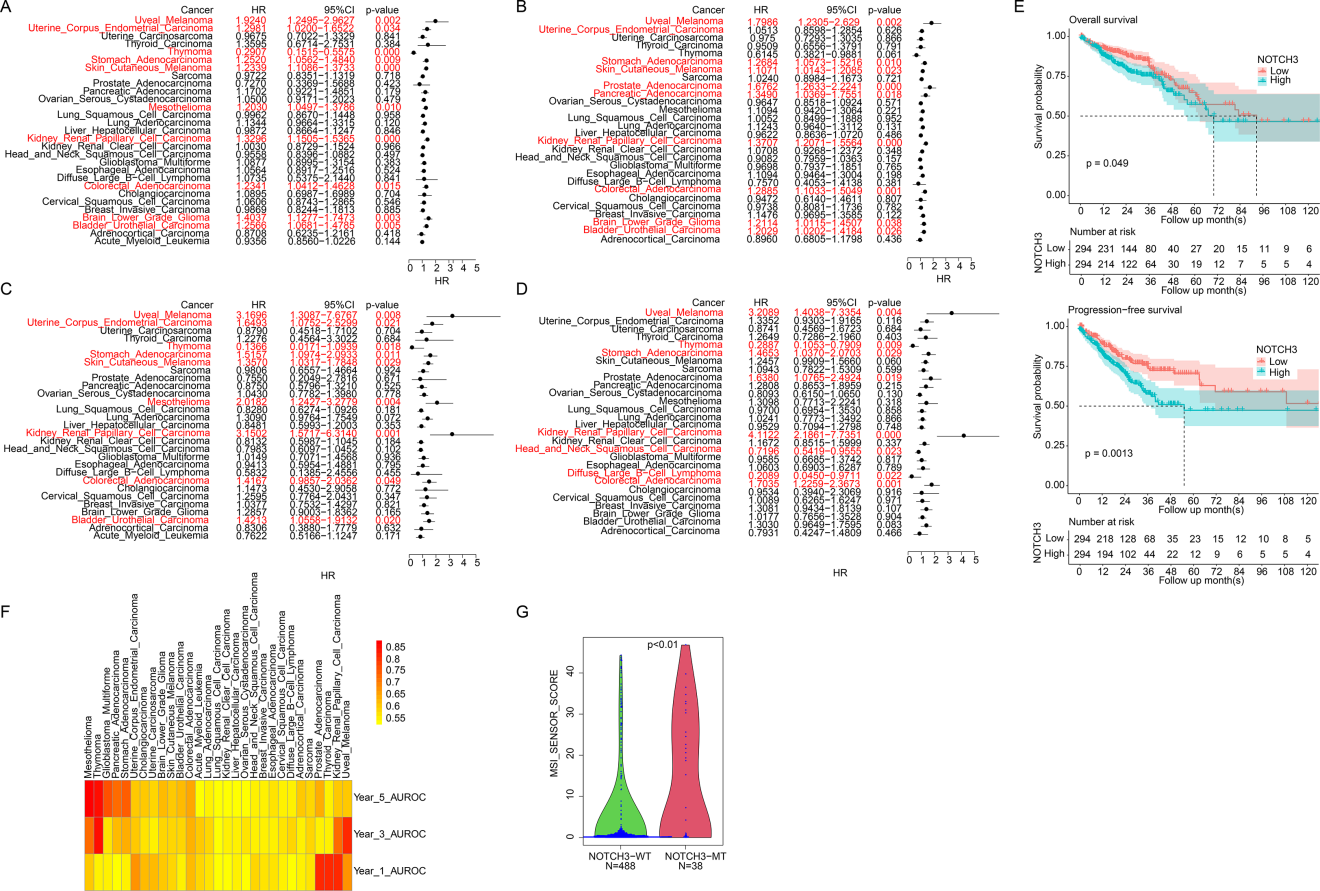
NOTCH3 expression predicts poor survival across cancers. **(a)** Forest plots of Cox regression for NOTCH3 association with **(a)** overall survival (OS) and **(b)** progression-free survival (PFS) in multiple cancers (HR >1 indicates worse prognosis) using Cox univariate regression. **(c, d)** Comparing high vs. low NOTCH3 expression groups for **(c)** OS and **(d)** PFS, showing pvalue and HR. Sample size: Acute Myeloid Leukemia: 161; Adrenocortical Carcinoma: 78; Bladder Urothelial Carcinoma: 406; Brain Lower Grade Glioma: 513; Breast Invasive Carcinoma: 1082; Cervical Squamous Cell Carcinoma: 294; Cholangiocarcinoma: 36; Colorectal Adenocarcinoma: 588; Diffuse Large B-Cell Lymphoma: 48; Esophageal Adenocarcinoma: 181; Glioblastoma Multiforme: 154; Head and Neck Squamous Cell Carcinoma: 514; Kidney Renal Clear Cell Carcinoma: 510; Kidney Renal Papillary Cell Carcinoma: 282; Liver Hepatocellular Carcinoma: 365; Lung Adenocarcinoma: 501; Lung Squamous Cell Carcinoma: 478; Mesothelioma: 86; Ovarian Serous Cystadenocarcinoma: 299; Pancreatic Adenocarcinoma: 177; Prostate Adenocarcinoma: 493; Sarcoma: 253; Skin Cutaneous Melanoma: 426; Stomach Adenocarcinoma: 407; Thymoma: 118; Thyroid Carcinoma: 497; Uterine Carcinosarcoma: 57; Uterine Corpus Endometrial Carcinoma: 526; Uveal Melanoma: 80. **(e)** Overall (top) and progression-free (bottom) survival curve of NOTCH3-low and NOTCH3-high samples in TCGA-CRC dataset. The bottom represents the cancer types (N = 588). **(f)** Area under the receiving operating characteristic (AUROC) curves for 1/3/5-year survival prediction using NOTCH3 expression across cancers. (Same sample size as A-D) **(g)** MSI score differences between NOTCH3 mutant vs. wild-type CRC.

We next analyzed the impact of NOTCH3 on clinical outcomes across different cancers. The prognostic value of NOTCH3 expression for overall and progression-free survival was assessed using Cox univariate regression. Elevated NOTCH3 expression predicted poor overall survival in uveal melanoma, skin melanoma, uterine cancer, gastric cancer, mesothelioma, kidney renal papillary cell carcinoma, colorectal cancer, lower-grade glioma, and bladder cancer, while opposite in thymoma (**Figure 2a**). A similar trend was observed for progression-free survival (**Figure 2b**). Patients in each cancer type were equally divided into high- and low-expression groups based on NOTCH3 expression in the corresponding cohort, and survival differences between the groups were evaluated. The high-NOTCH3 expression group exhibited worse overall and progression-free survival (**Figures 2c, d**), which is similar to Cox univariate regression. The consistent findings demonstrate the prognostic value of NOTCH3, which is robust in specific cancer types—regardless of the statistical algorithms used—yet exhibits heterogeneity across cancer types. Specifically, NOTCH3 mRNA abundance was significantly correlated with both overall and progression-free survival in both Cox regression (**Figures 2a, b**) and group comparison analyses (**Figures 2c–e**) in CRC. The predictive performance of NOTCH3 was further evaluated using the area under the receiver operating characteristic curve for one-, three-, and five-year survival across cancer types (**Figure 2f**). NOTCH3 expression has a favorable predictive value in several cancers. We also examined the impact of its somatic mutations on survival. However, NOTCH3 mutations in most cancers were not significantly associated with overall survival (**Supplementary Figure S1**; N = 488 for wild-type, N = 38 for mutant), possibly due to limited NOTCH3-mutated sample, or it may influence the prognosis under other conditions (i.e treatment). Furthermore, NOTCH3-mutated cases showed significantly higher microsatellite instability (MSI) scores than wild-type cases (**Figure 2g**). In summary, NOTCH3 expression serves as a prognostic indicator in multiple cancer types, including CRC.

### NOTCH3 mutations affect the immune infiltration in pancancer and CRC

**Figure 3:**
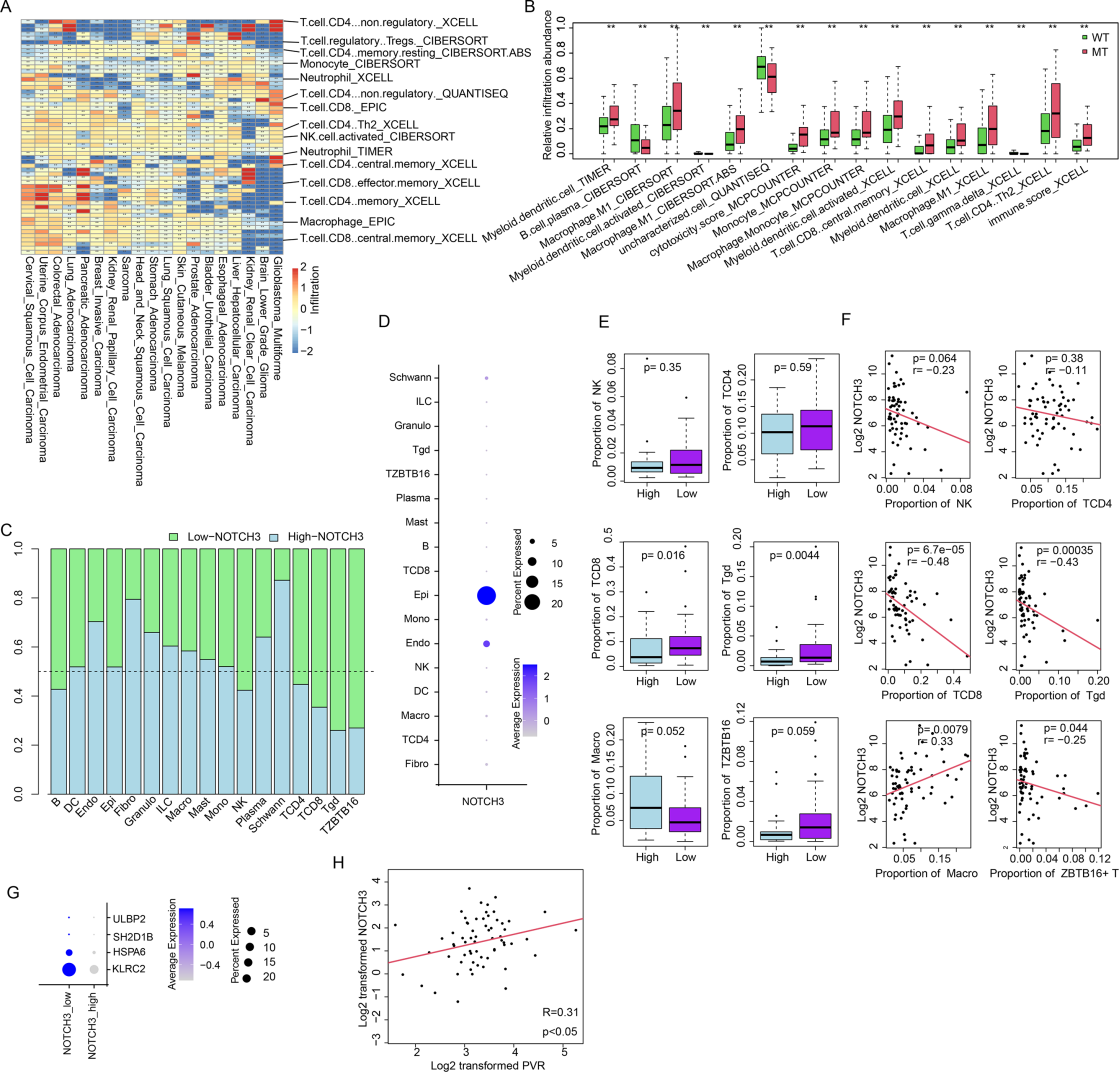
NOTCH3 alteration enhance immune infiltration in CRC. **(a)** Heatmap of immune cell abundance differences in NOTCH3-mutant vs. wild-type tumors across cancers. Blue corresponds to diminished infiltration in NOTCH3-mutated tumors and red represent increased. Only the representative cell types indicated in the right part of the figure. Sample size sued listed as below: Acute Myeloid Leukemia: 174, Adrenocortical Carcinoma: 79, Bladder Urothelial Carcinoma: 408, Brain Lower Grade Glioma: 515, Breast Invasive Carcinoma: 1083, Cervical Squamous Cell Carcinoma: 295, Cholangiocarcinoma: 37, Colorectal Adenocarcinoma: 593, Diffuse Large B-Cell Lymphoma: 49, Esophageal Adenocarcinoma: 182, Glioblastoma Multiforme: 161, Head and Neck Squamous Cell Carcinoma: 516, Kidney Renal Clear Cell Carcinoma: 511, Kidney Renal Papillary Cell Carcinoma: 284, Liver Hepatocellular Carcinoma: 367, Lung Adenocarcinoma: 511, Lung Squamous Cell Carcinoma: 485, Mesothelioma: 88, Ovarian Serous Cystadenocarcinoma: 301, Pancreatic Adenocarcinoma: 178, Prostate Adenocarcinoma: 494, Sarcoma: 254, Skin Cutaneous Melanoma: 444, Stomach Adenocarcinoma: 413, Thymoma: 120, Thyroid Carcinoma: 499, Uterine Carcinosarcoma: 58, Uterine Corpus Endometrial Carcinoma: 528, Uveal Melanoma: 81 **(b)** Immune infiltration difference in CRC (boxplot showing infiltration abundances in wild-type and mutant samples). **(c)** Normalized cell proportion difference between NOTCH3-high vs. -low groups. **(d)** Expression of NOTCH3 across cell types, where dot size represents percentage of cells expression NOTCH3 and the color indicates average expression value. **(e)** Immune cell proportion difference between NOTCH3-low and NOTCH3-high samples. **(f)** Correlation between NOTCH3 expression and immune cell proportions. The x-axis is the cell proportion and y-axis is log2 transformed NOTCH3 expression **(g)** Expression of NK and CD8+T cell marker in NOTCH3-low and NOTCH3-high samples in scRNA-seq data. **(h)** PVR expression and NOTCH3 expression in scRNA-seq samples, each dot represents a sample (N = 62), corresponding to the expression of PVR (x axis) and NOTCH3 (y-axis). **p < 0.01.

The tumor microenvironment, shaped by cell–cell interactions, plays a critical role in CRC development and progression. Therefore, we investigated the association between NOTCH3 and immune infiltration. After estimating immune cell abundances using different algorithms, we compared immune infiltration proportions between NOTCH3-mutant and wild-type samples (**Figure 3a**). NOTCH3 mutations were significantly associated with altered infiltration of various immune cells. In CRC, NOTCH3 mutation was characterized by increased proportions of central memory CD8^+^ T cells and M1 macrophages (**Figure 3b**). Cytotoxicity and immune scores were also higher in NOTCH3-mutant samples. The immune infiltration pattern in NOTCH3-low and NOTCH3-high expression samples resembled NOTCH3 mutation (**Supplementary Figure S2**). We further examined infiltration differences between NOTCH3-low and NOTCH3-high expression samples using CRC single-cell RNA sequencing data. After cell type annotation, cell proportions were analyzed (**Figure 3c**). NOTCH3-high samples showed a lower proportion of T cells including ZBTB+ T cell (19), CD8+T cell and γδ T cells (**Figure 3c**). Interestingly, total macrophage proportion was higher. NOTCH3 was primarily expressed in epithelial (cancer) cells (**Figure 3d**). The cell proportions of NOTCH3-low and NOTCH3-high samples were compared. CD8^+^ T cells and γδ T cells were more abundant in NOTCH3-low than in NOTCH3-high samples (**Figure 3e**). We also assessed the Spearman correlation between NOTCH3 expression and immune cell proportions. As expected, NOTCH3 expression was significantly negatively correlated with CD8^+^ T cells, γδ T cells, and ZBTB16^+^ T cells, but positively correlated with total macrophages (**Figure 3f**). In addition, CD8^+^ T cells from NOTCH3-low samples showed higher expression of KLRC2, HSPA6, and SH2D1B—markers associated with NK cells and CD8^+^ T cells—compared to those from NOTCH3-high samples (20) (**Figure 3g**). PD-L1 and PVR are key genes expression on cancer cells to inhibit the activity of CD8+ T cells, NK cells and γδ T cells. Thus, the correlation between NOTCH3 and these genes were also analyzed. Positive correlation of NOTCH3 and PVR expression in the scRNA-seq data (**Figure 3h**) was detected, but not PD-L1 (not shown). In summary, NOTCH3 expression is significantly associated with effector immune cell infiltration (CD8^+^ T and γδ T cells) and PVR expression.

### PVR-TIGIT interaction in NOTCH3-high CRC samples

**Figure 4:**
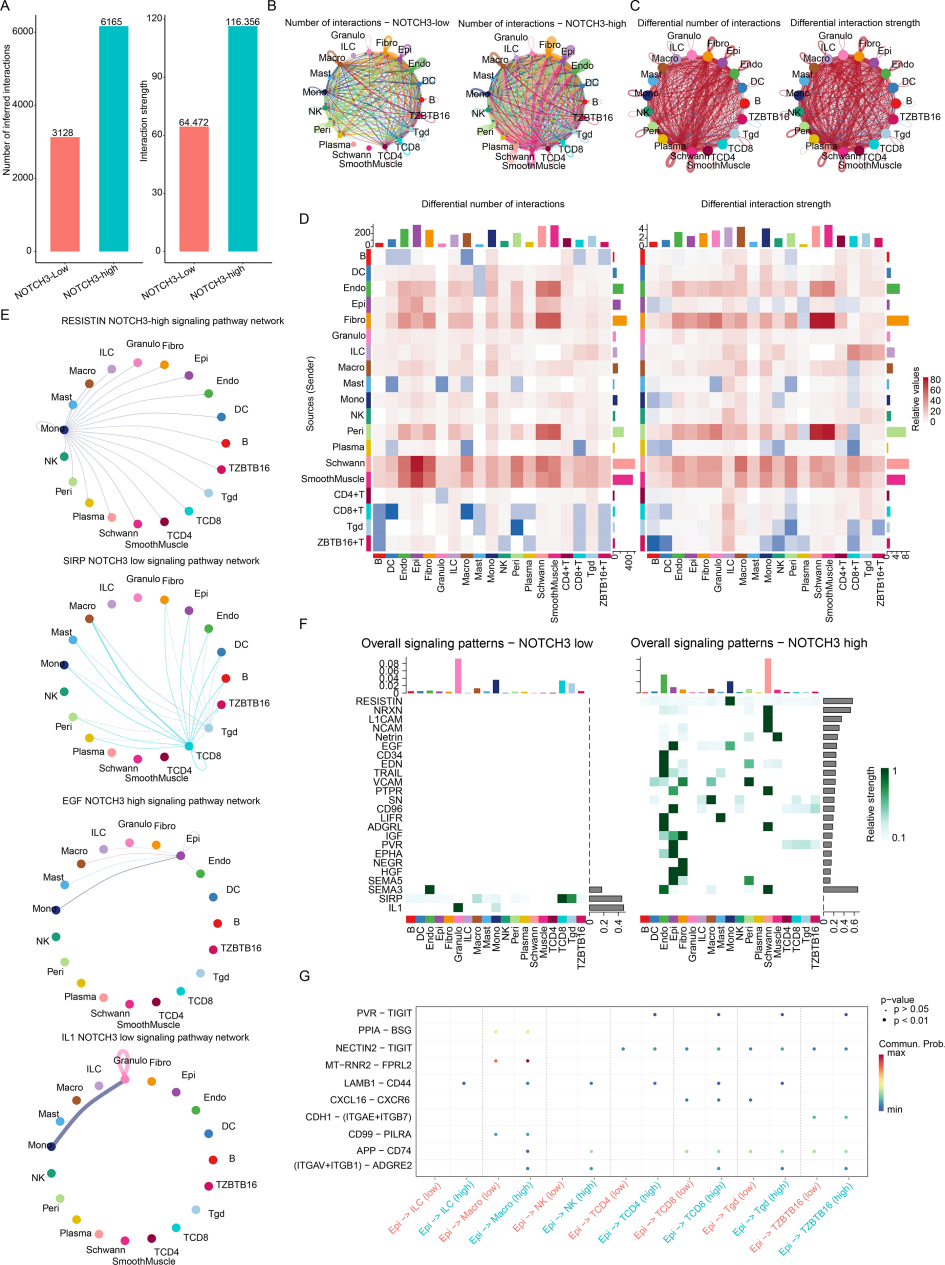
NOTCH3 regulates PVR-mediated cell-cell interactions. **(a)** Total interaction number and strength comparison of NOTCH3-low and NOTCH3-high samples. **(b)** Overall interaction pattern of NOTCH3-low and NOTCH3-high samples. **(c, d)** Differential cell interactions (NOTCH3-high vs NOTCH3-low): **(c)** network diagrams and **(d)** heatmap. NOTCH3-low and NOTCH3-high specific pathways, displayed in **(e)** network and **(f)** heatmap. **(g)** Bubble plot showing the interaction pathways between cancer cells and immune cells (ILC, NK, CD4+ T, CD8+ T, γδ T and ZBTB16+ T cells), highlighting PVR pathway in NOTCH3-high samples.

We next investigated the impact of NOTCH3 on cell–cell interactions in CRC using scRNA-seq data. Overall, both the number and strength of cell–cell interactions were significantly greater in NOTCH3-high samples compared to NOTCH3-low samples (**Figure 4a**). Most interactions between cell types were present in both NOTCH3-low and NOTCH3-high samples (**Figure 4b**), though their interaction strengths and frequencies differed (**Figures 4c, d**). It is noted that Interactions involving CD8^+^ T cells and ZBTB1+ T cells were decreased in NOTCH3-high samples compared to NOTCH3-low samples (**Figures 4c, d**). In addition, we identified signaling pathways specifically detected in either NOTCH3-low or NOTCH3-high samples, including RESISTIN, SIRP, EGF, and IL1 pathways (**Figures 4e, f**). The PVR pathway was detected in NOTCH3-high samples but absent in NOTCH3-low samples (**Figure 4f**). Since effector cells directly mediate cytotoxicity against cancer cells, we analyzed interactions between cancer cells (epithelial) and natural killer cells, CD8^+^ T cells, macrophages, and γδ T cells. Although their most interaction patterns were similar, the PVR–TIGIT interaction was detected at significant level only in NOTCH3-high samples (**Figure 4g**). Taken together, NOTCH3 expression remarkably associated with cell-cell interaction profile, particularly interactions between cancer cells and CD8^+^ T cells via the PVR pathway.

### NOTCH3 modulates CD8+T cytocytoxicies via PVR in CRC

**Figure 5:**
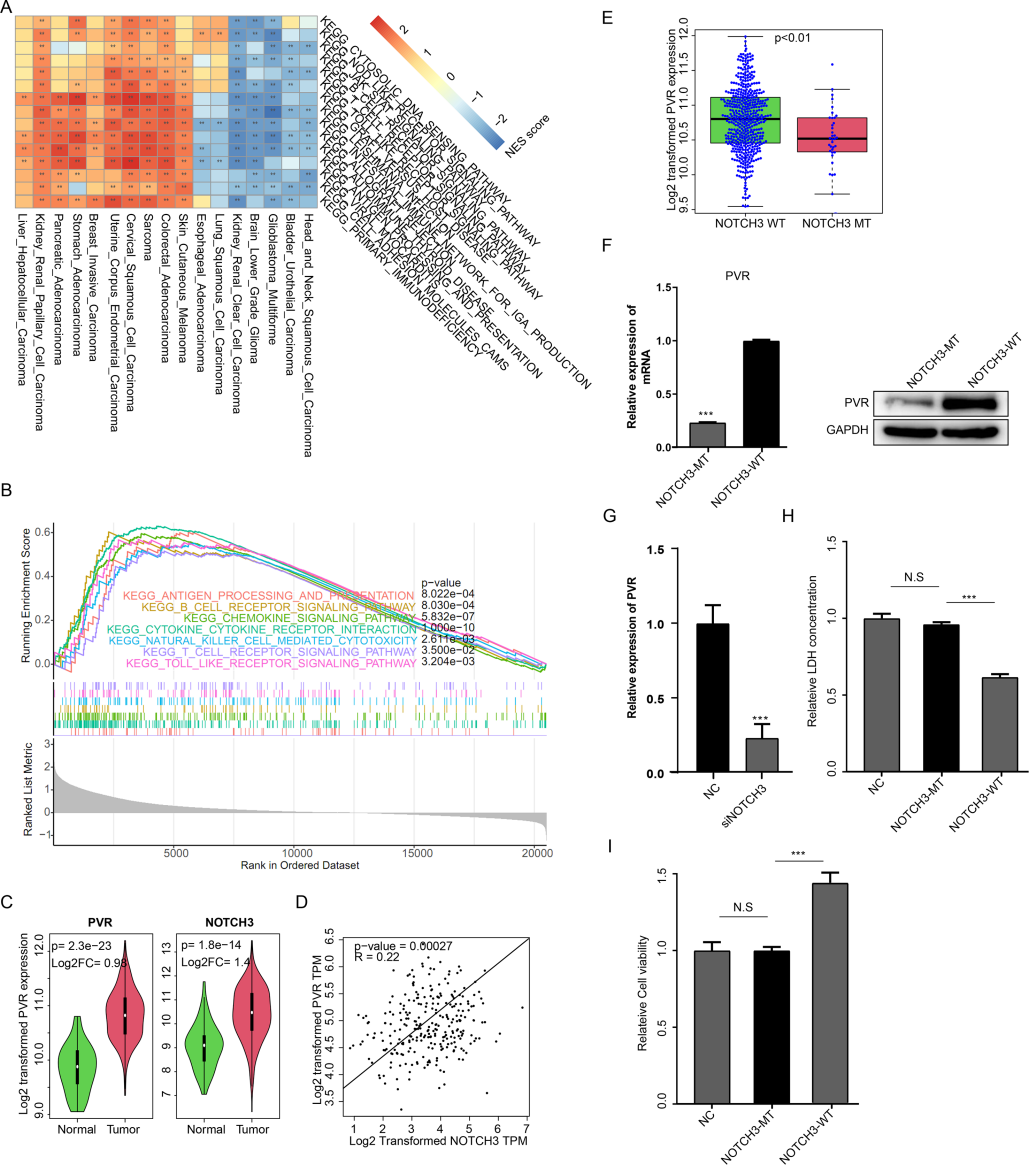
NOTCH3 impairs CD8+ T cell cytotoxicity via PVR. **(a)** Heatmap of normalized enrichment score (NES) of immune-related KEGG pathways by comparing the transcriptome of NOTCH3-WT and NOTCH3-MT tumors, using GSEA. Red represents elevated and blue is decreased. Bladder Urothelial Carcinoma: 408, Brain Lower Grade Glioma: 515, Breast Invasive Carcinoma: 1083, Cervical Squamous Cell Carcinoma: 295, Colorectal Adenocarcinoma: 593, Esophageal Adenocarcinoma: 182, Glioblastoma Multiforme: 161, Head and Neck Squamous Cell Carcinoma: 516, Kidney Renal Clear Cell Carcinoma: 511, Liver Hepatocellular Carcinoma: 367, Lung Adenocarcinoma: 511, Lung Squamous Cell Carcinoma: 485, Mesothelioma: 88, Pancreatic Adenocarcinoma: 178, Prostate Adenocarcinoma: 494, Sarcoma: 254, Skin Cutaneous Melanoma: 444, Stomach Adenocarcinoma: 413, Uterine Corpus Endometrial Carcinoma: 528. **(b)** Immune-related pathway activation in NOTCH3-mutant CRC samples. **(c)** NOTCH3 and PVR expression was enhanced cancer samples compared to the normal in TCGA-CRC dataset.**(d)** PVR expression is correlated with NOTCH3 expression. **(e)** NOTCH3 mutated TCGA-CRC samples express lower PVR. **(f)** PVR expression was reduced in NOTCH3-mutant samples compared to NOTCH3-wild type. **(g)** PVR expression was reduced in NOTCH3 knocking down samples compared to the control. CD8+ T (primary cells isolated from whole blood) cytotoxicity assays: **(h)** LDH release and **(i)** cell viability (N.S p>0.05, ***p<0.001).

We employed Gene Set Enrichment Analysis (GSEA) to assess the pathway-level differences between NOTCH3-wild-type and NOTCH3-mutant samples from The Cancer Genome Atlas (TCGA, **Figure 5a**). In most cancer types, NOTCH3 mutations were associated with elevated immune-related pathways, including the T cell receptor, B cell receptor, antigen processing and presentation pathways, and natural killer cell-mediated cytotoxicity in most cancers; this was also observed in CRC (**Figure 5b**). Our previous results have shown that NOTCH3 alterations (expression and mutation) are associated with survival, immune infiltration, and CD8^+^ T cell-related PVR pathways. Furthermore, scRNA-seq data revealed that the PVR pathway activity was significantly different in NOTCH3-low and NOTCH3-high samples. Thus, we hypothesized that NOTCH3 may regulate PVR expression to modulate CD8^+^ T cell cytotoxicity in CRC, as PVR is a classical immune checkpoint pathway that regulates CD8^+^ T cell activity (21). Both NOTCH3 and PVR expression were elevated in CRC compared to normal tissue (**Figure 5c**). PVR expression was significantly correlated with NOTCH3 expression in TCGA dataset (**Figure 5d**). Moreover, NOTCH3-mutant CRC samples showed significantly lower PVR levels in the TCGA dataset (**Figure 5e**). NOTCH3 R1669H mutation (the most frequent mutation site in the TCGA dataset, **Figure 1b**, **Supplementary Figure S3**) significantly reduced PVR expression compared to the wild-type (**Figure 5f**). We knocked down NOTCH3 in HCT116 cells and found that PVR expression was downregulated (**Figure 5g**). We then assessed CD8^+^ T cell cytotoxicity using an LDH cytotoxicity assay. When co-cultured with HCT116 cells overexpressing NOTCH3-WT, CD8^+^ T cell cytotoxicity was significantly decreased (**Figure 5h**). However, no significant difference in CD8^+^ T cell cytotoxicity was detected in the NOTCH3-MT group compared to the control. Cell viability analysis yielded results consistent with the LDH assay (**Figure 5i**). Knocking down PVR restored the effect of NOTCH3 (**Supplementary Figure S4**). In conclusion, NOTCH3 upregulates PVR expression and reduces CD8^+^ T cell cytotoxicity, while its mutation (R1669H) disrupts this function.

### NOTCH3 regulates PVR expression by targeting RBPJ in CRC

**Figure 6:**
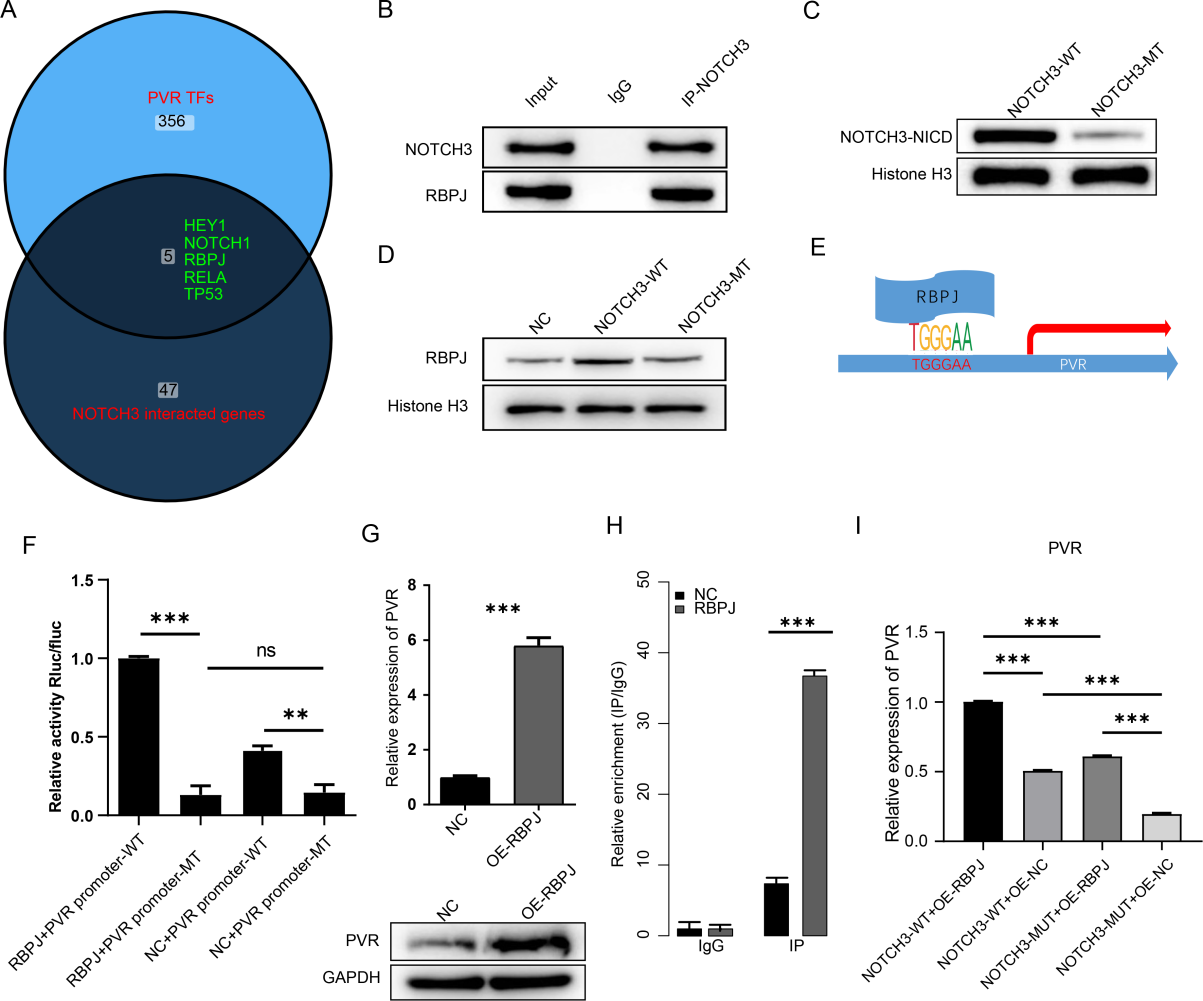
NOTCH3-RBPJ axis transcriptionally activates PVR. **(a)** Venn diagram of PVR regulators intersecting NOTCH3 interacted proteins and transcription factors (TFs) of PVR. **(b)** Co-IP showing NOTCH3-RBPJ binding. **(c)** Nuclear NOTCH3 accumulation with WT vs. MT (subcellular fractionation). **(d)** Nuclear RBPJ accumulation with WT vs. MT (subcellular fractionation). **(e)** PVR promoter region has a RBPJ binding motif. **(f)** Luciferase reporter assay showing the activity of PVR promoter for RBPJ. **(g)** PVR induction by RBPJ overexpression (qPCR and Western blotting). **(h)** ChIP-PCR examing RBPJ binding to PVR promoter, using IgG and RBPJ antibody. **(i)** Synergistic PVR activation by NOTCH3+RBPJ. (n.s p>0.05, **p<0.01, ***p<0.001).

Since the activation of canonical NOTCH pathway initiates expression of downstream genes, we suspect that NOTCH3 also regulates PVR expression by binding to transcription factors. We screened the predicted transcription factors of PVR using the TFlink database (22) and NOTCH3-interacting genes from the STRING database (23). Five genes were identified in the intersection (**Figure 6a**), including HEY1, NOTCH1, RBPJ, RELA, and TP53. Among these genes, RBPJ is a classical downstream effector of the NOTCH family. Thus, we assayed whether NOTCH3 promotes the transcription of PVR via RBPJ. The co-immunoprecipitation (co-IP) assay revealed that NOTCH3 binds to RBPJ in HCT116 cell line (**Figure 6b**). Transfection of NOTCH3-WT significantly increased nuclear NOTCH3 levels compared to NOTCH3-MT (**Figure 6c**). Consistently, the overexpression NOTCH3-WT significantly also enhanced the nucleus RBPJ level, while not NOTCH3-MT (**Figure 6d**), suggesting that NOTCH3 binds to RBPJ and facilitate RBPJ for nucleus translocation. NOTCH3-WT overexpression also up-regulated the canonical downstream gene HEY1 and HES1 (**Supplementary Figure S5**) relative to NOTCH3-MT. We next analyzed whether RBPJ binds to the promoter region of PVR and initiates its transcription, as a transcription factor. The binding motif was retrieved from the JASPAR database and PVR promoter harbors a binding site for RBPJ (**Figure 6e**). Luciferase assay revealed that introduction of the wild-type PVR promoter region significantly enhanced luciferase activity in presence of RBPJ, while the mutant promoter showed no significant difference (**Figure 6f**). Meanwhile, overexpression of RBPJ also enhanced the expression level of PVR, on both mRNA and protein levels (**Figure 6g**). In addition, ChIP-PCR revealed that the PVR promoter region was significantly enriched in the presence of RBPJ (**Figure 6h**) compared to IgG. Furthermore, the presence of both RBPJ and NOTCH3-WT significantly enhanced PVR expression levels (**Figure 6i**). Knocking down of RBPJ significantly reduced the NOTCH3 induced PVR up-regulation (**Supplementary Figure S6**). In summary, NOTCH3 interacts with RBPJ, facilitate its nucleus translocation. RBPJ binds to the promoter of PVR and initiates PVR expression.

### NOTCH3 inhibition facilitates immunotherapy in CRC

**Figure 7:**
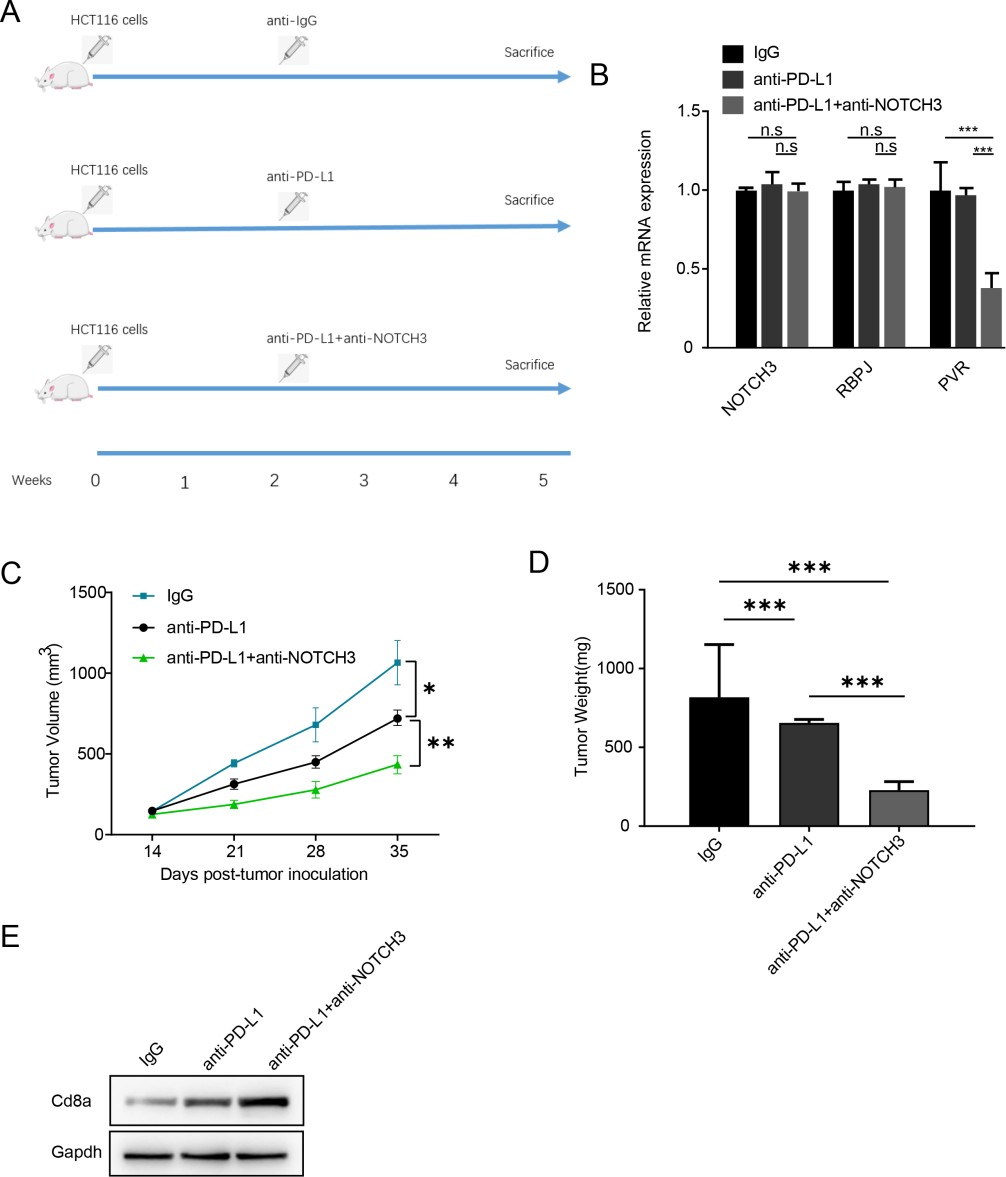
NOTCH3 mutation enhances anti-PD-L1 response *in vivo*. **(a)** Processing steps of different grouped mice (N = 5 for each group). **(b)** NOTCH3, RBPJ and PVR expression in the IgG, anti-PD-L1 and anti-PD-L1+anti-NOTCH3 groups estimated by qRT-PCR. **(c)** Tumor volume overtime of different groups. **(d)** Tumor weight at sacrifice of different groups. **(e)** Cd8a abundance in three groups evaluated using Western blotting (n.s not significant, *p<0.05, **p<0.01, ***p<0.001).

Since the PVR–TIGIT interaction is a key immune checkpoint for CD8^+^ T cell cytotoxicity, and the goal of immunotherapy is to activate CD8^+^ T cells by inhibiting PD-1/PD-L1/CTLA4 (also checkpoint genes), we investigated whether NOTCH3 blockade enhances the efficacy of immunotherapy in CRC. To test this hypothesis, we injected the CRC cell line HCT116 into C57BL/6 mice (proficient immune system) and treated them with IgG, anti-PD-L1, and/or anti-NOTCH3 antibody (**Figure 7a**; n = 5 per group). Tumor size was evaluated after 5 weeks. Anti-NOTCH3 antibody did not alter the mRNA expression of NOTCH3 or RBPJ as expected, while significantly reduced PVR expression *in vivo* (**Figure 7b**). Compared to the IgG group, anti-PD-L1 therapy reduced tumor size and volume. Moreover,​the combination of anti-PD-L1 and anti-NOTCH3 further enhanced the effect of anti-PD-L1 therapy (**Figures 7c, d**), on both tumor weight and volume. Consistently, Cd8a expression (marker for CD8+ T cell) was significantly increased in the combination group compared to single PD-L1 treatment (**Figure 7e**). In summary, NOTCH3 depletion enhances the efficacy of anti–PD-L1 treatment in a CRC mouse xenograft model.

### NOTCH3 predicts clinical outcome of immunotherapy treated CRC patients

**Figure 8:**
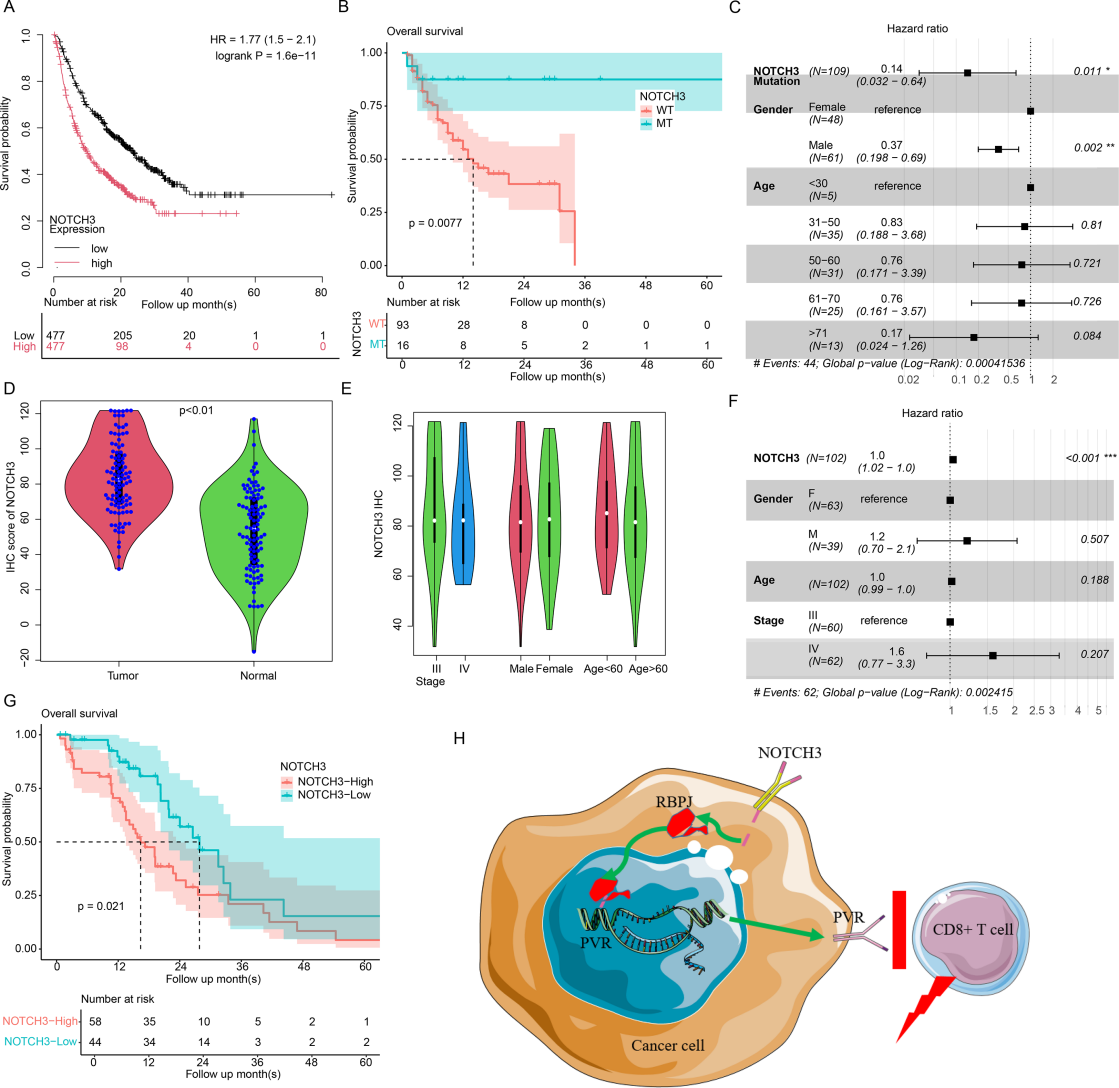
Clinical validation of NOTCH3 as an immunotherapy biomarker. **(a)** Survival difference between NOTCH3-low and NOTCH3-high pan-cancer patients who received immunotherapy (N = 954). **(b)** Survival difference between NOTCH3-wild-type and NOTCH3-mutant patients who received immunotherapy in MSKCC-CRC dataset (N = 109). **(c)** Multivariate Cox regression of for overall survival using NOTCH3 mutation and other clinical indicators in MSKCC dataset (N = 109). **(d)** NOTCH3 IHC in tumor vs. normal in the samples we collected (N = 102). **(e)** Clinical signatures associated with NOTCH3 expression. **(f)** Multivariate Cox regression of for overall survival using NOTCH3 mutation and other clinical indicators in our dataset (N = 102). **(g)** Kaplan-Meier analysis by NOTCH3 IHC levels (high vs. low; log-rank p<0.05, N = 102). **(h)** The mechanism of NOTCH3. n.s, not significant, *p < 0.05, **p < 0.01, ***p < 0.001.

We also analyzed the impact of NOTCH3 alterations (expression and mutation) on immunotherapy outcomes. Using the TISIDB database (a pan-cancer cohort of immunotherapy-treated patients), we compared survival between NOTCH3-high and NOTCH3-low patients (**Figure 8a**, N = 954). NOTCH3-low patients showed better survival than NOTCH3-high patients. We also evaluated survival differences in an independent cohort of 109 immunotherapy-treated patients (16 with NOTCH3 mutations) from the MSKCC immunotherapy cohort, comparing NOTCH3-mutant and NOTCH3-wild-type groups. The NOTCH3-mutant group demonstrated significantly prolonged survival compared to the wild-type group (**Figure 8b**, p<0.01). Cox multivariate regression confirmed NOTCH3 status as an independent prognostic factor (**Figure 8c**). We also retrospectively analyzed 102 immunotherapy-treated patients from our institution and other centers. NOTCH3 expression was quantified using immunohistochemistry on tissue microarrays, which revealed overexpression in tumor tissue compared to normal tissue (**Figure 8d**), consistent with previous findings. Although NOTCH3 expression was not significantly associated with clinical indicators such as age, sex, or stage (**Figure 8e**), Cox multivariate regression also identified it as an independent predictor of survival in immunotherapy-treated CRC patients (**Figure 8f**). When patients were stratified into NOTCH3-high and NOTCH3-low groups, the NOTCH3-high group showed significantly worse survival (**Figure 8g**). Together, these results demonstrate that NOTCH3 serves as a predictive biomarker for immunotherapy response in CRC.

## Material and method

### Sample enrollment, data retrieval and processing

This study was approved by the Ethics Committee of Henan Cancer Hospital conducted under Declaration of Helsinki. We enrolled CRC patients hospitalized at our institution between 2014 and 2022 in this study. Written informed consent was obtained from all participating individuals. All samples involved are MSI CRC according to the treatment guideline, using Candonilimab (dual anti-PD-1/CTLA-4). In the MSKCC dataset, the samples are also mostly MSI, with differed treatment drugs (anti-PD-(L1) and/or anti-CTLA4). Multiple ‘omics’ data and phenotype data was obtained from the cBioPortal database (24). Gene expression data were log2-transformed, and synonymous mutation data were filtered from the genomic data. For single-cell sequencing data, raw count data from GSE178341 (19) was downloaded via the Gene Expression Omnibus (GEO) database along with the annotated information. In this analysis, only tumor samples were used. Samples originating from different sections of the same patient were treated as distinct samples.

### Single cell sequencing and cell-cell interaction analyses

After data retrieval and pre-processing, quality control was performed using the R package ‘Seurat’ (25). Cells were clustered and annotated according to previously reported methods. Pseudo-bulk RNA-seq was performed using Seurat::AggregateExpression to generate a raw count matrix. After transforming the raw count matrix to counts per million (CPM) values, samples were stratified into NOTCH3-low and NOTCH3-high cohort based on the median CPM value of NOTCH3. Cell proportions were calculated according to the number of each cell type in the corresponding samples. Cell proportion differences were evaluated using the Wilcoxon test. The correlation of NOTCH3 CPM values and cell proportions was calculated using Pearson correlation. For cell-cell interaction analyses, the interaction number and strength between NOTCH3-low and NOTCH3-high samples were compared using the R package “CellChat” (26), following the recommended steps and default parameters.

### Bioinformatics analyses (Infiltration and GSEA)

For immune cell infiltration estimation, log2-transformed TPM values from the TCGA dataset were analyzed using the TIMER2 website (27) which incorporates multiple estimation algorithms (CIBERSORT (28), XCELL (29), QUANTISEQ (30), etc.), yielding a pan-cancer immune infiltration matrix. Infiltration differences between groups were compared. For GSEA analyses, samples for each cancer type were stratified into NOTCH3 wild-type (NOTCH3-WT) and NOTCH3 mutant (NOTCH3-MT) groups based on non-synonymous mutation status. Log2-transformed fold changes and corresponding p-values between groups were calculated. Following gene ranking by log2 fold change, Gene Set Enrichment Analysis (GSEA) was carried out and visualized utilizing R packages ‘clusterProfiler’ (31).

### Overexpression plasmid construction

To construct a NOTCH3 (R1669H) mutant (NOTCH3-MT) overexpression plasmid, we first obtained a wild-type (WT) NOTCH3 overexpression plasmid (pcDNA3.1) as a template. Complementary primers for site-directed mutagenesis were designed with the mutation centered within 35 bp of the primer sequences. Amplification was implemented with DNA polymerase. The product was then transformed into competent E. coli cells, plated on antibiotic-containing media, and positive colonies were screened. The successful introduction of the R1669H mutation was confirmed by Sanger sequencing of the target region, and plasmid integrity was verified through restriction enzyme digestion analysis. For functional studies, the confirmed mutant plasmid was transfected into target cells for expression analysis.

### Western blotting, RNA isolation and qRT-PCR

For WB, cells were lysed and centrifuged, 150 μL of Dilute Buffer was added to the tube, which was vortexed, incubated for 5 min, centrifuged (12,000, 15 min, 4 °C). The clarified supernatant (500 μL) was decanted into new tubes, mixed with 500 μL of pre-cooled isopropanol, and vigorously vortexed for 15 s, and incubated for 10 min. It was centrifuged, and the upper phase was removed. It was washed using pre-cooled 75% ethanol, mixed 5 times, and centrifuged. The supernatant was discarded, and the pellet was washed again with 75% ethanol. The RNA pellet was dried in a 37 °C heating block for 2 min and dilute to ~500 ng/μL, followed by additional drying at 37°C for 5 min. RNA concentration was measured. A 1% agarose gel was prepared, and 4 μL of 1 kb Plus DNA ladder was loaded. Electrophoresis was performed at 120 V for 25 min. Total RNA (1 μg) was reverse transcribed, and 140 μL of nuclease-free water was added. Primers were diluted to 10 μM. qPCR was performed to quantify reference and target genes, and gene expression value was normalized based on the endogenous control.

For protein analysis, separating and stacking gels were prepared, loaded, and electrophoresed at 80 V, followed by an increase to 120 V. The gel was rinsed, and wet transfer was performed at 80 V in ice-water for 1.5–2 hours. The PVDF membrane was removed and stained with Ponceau S, and washed. Blocking was performed with 20 mL of 5% BSA for 1.5 h. After removal the solution, the membrane was briefly washed. BSA diluted primary antibody was added and incubated for 2 hours. Secondary antibody diluted in 5% BSA was added and incubated for 1.5 h. The secondary antibody was discarded, and the membrane was placed in a washing box, rinsed three times with 20 mL of 1× TBST (15 min per wash). The membrane was then transferred to a chemiluminescence imager, and luminescent signals were acquired after adding the substrate.

### ChIP-PCR and luciferase assay

For luciferase assays, cells were properly seeded in medium. For transfection, 1 µL of TSnanofect V1 was mixed with 49 µL of antibiotic-free, serum-free medium or PBS to prepare a 50 µL solution, which was then combined with 50 µL of plasmid DNA and incubated for 5 minutes. Subsequently, 100 µL of the transfection mixture was added to each well, and the total volume was adjusted to 500 µL with fresh medium. After 48 hours of incubation at 37°C, gene expression levels were analyzed. The culture medium was aspirated, and cells were lysed by adding 100 µL of passive lysis buffer (PLB), followed by gentle agitation for 15 minutes. A 20 µL aliquot of cell lysate was transferred to a luminometer tube for luciferase activity measurement. Firefly luciferase luminescence signals were recorded, after which 100 µL of Stop & Glo^®^ reagent was used to assay Renilla luciferase activity.

For ChIP-PCR, cells were cultured to appropriate confluence, and PCR primers were designed to flank the RBPJ binding sites. Following the provided manual for the Simple ChIP^®^ Enzymatic Chromatin IP Kit (Agarose Beads) (Cell Signaling, USA), cells were cross-linked using formaldehyde, and chromatin was prepared. Nuclei were isolated and chromatin was enzymatically digested to optimal fragment sizes. Chromatin was immunoprecipitated overnight with specific antibodies bound to protein G agarose beads. After elution and reversal of cross-links, DNA was purified and verified with agarose gels.

### Mice model

The Animal Care and Use Committee of The Affiliated Cancer Hospital of Zhengzhou University has approved this work. Immune proficient male mice (5 weeks, ​C57BL/6) mice were acquired from Shanghai Model Organisms and housed in the experimental animal center of our institution. Mice were subcutaneously implanted with HCT116 CRC cells, followed by treatment with anti-PD-L1 antibody (10 mg/kg, administered every other day; Bio X Cell, USA) and/or NOTCH3 antibody (10mg/kg, 55114-1-AP, injected every the other day, proteintech, China) beginning two week post-injection. Upon completion of the treatment protocol, all animals were humanely euthanized via controlled CO2 asphyxiation at a flow rate of 30% of the chamber volume per minute (AVMA guideline) to facilitate surgical resection of xenograft tumors for subsequent analysis.

### LDH assay and IHC

For NOTCH3 quantification, tissue microarrays (TMAs) were generated internally following established protocols. Immunohistochemical analysis was performed on 7-μm tissue sections obtained from formalin-fixed paraffin-embedded tissue. Initial processing included sequential deparaffinization and rehydration using a graded ethanol series. After antigen retrieval, peroxidase was blocked with 3% H_2_O_2_ for 20 minutes. Primary antibody against NOTCH3 was applied and incubated at 4 °C overnight. Sections were then incubated with secondary antibodies, and three 5-minute washes with PBS. Diaminobenzidine (DAB) substrate (5 mL) was applied for 10 minutes for chromogenic development. Microscopic evaluation was conducted using a Leica DMRXA2 system with Leica Application Suite software. Staining intensity was quantified using ImageJ with the IHC Profiler plugin.

For LDH detection, cytotoxicity was measured in cell-free co-culture supernatants using the Lactate Dehydrogenase Cytotoxicity Assay Kit (Beyotime, C0016) fallowing the manual. NC (negative control), NOTCH3-WT, and NOTCH3-MT cells were co-cultured with CD8^+^T cells (IPHASE, 071A403.11). After 3 hours of co-culture, LDH concentration was determined following the manufacturer’s protocol.

### Statistics

We carried out all statistical analyses with R software (version 4.4.1). For comparisons between categorical and continuous variables, Student’s t-test and Wilcoxon rank-sum test were implemented according to variance homogeneity. Correlation analyses were conducted using Pearson correlation when parametric assumptions were satisfied; or Spearman’s rank correlation was applied. Three replicates were set for the experiments results. Throughout this study, a p-value < 0.05 was considered statistically significant.

## Discussion

This study elucidates a novel mechanism of NOTCH3-mediated immune evasion in colorectal cancer (CRC) through direct transcriptional activation of the immune checkpoint molecule PVR (**Figure 8h**). By integrating multi-omics analyses, functional validation, and preclinical modeling, we demonstrate that NOTCH3—frequently dysregulated in CRC and correlated with poor prognosis—orchestrates an immunosuppressive tumor microenvironment via the PVR-TIGIT axis. Mechanistically, we show that NOTCH3 physically interacts with RBPJ to bind the PVR promoter and drive its expression, while loss-of-function NOTCH3 mutations abrogate this regulation, thereby restoring CD8+ T cell cytotoxic activity. Single-cell resolution analysis revealed that NOTCH3-high tumors exhibit enhanced PVR-TIGIT interactions that selectively compromise CD8+ T cell function. Therapeutically, NOTCH3 depletion sensitized tumors to anti-PD-L1 therapy *in vivo*, paralleling clinical observations where NOTCH3 alterations predicted enhanced immunotherapy response. These findings address a fundamental gap in understanding NOTCH-mediated immune checkpoint regulation and propose a dual therapeutic strategy: targeting the NOTCH3-PVR axis in combination with PD-L1 blockade to overcome immune resistance in CRC. This approach holds particular promise for microsatellite-stable patients, who currently represent an unmet clinical need in CRC immunotherapy.

RBPJ is a canonical downstream effector of NOTCH signaling in cancer (32, 33). While its functions have been primarily characterized in macrophages and other immune cells (34), recent studies demonstrate its role in promoting T cell exhaustion in malignancies such as hepatocellular carcinoma (35–37). In contrast, the immunological impact of its upstream regulator NOTCH3 has been mainly documented in hematological malignancies like leukemia (38, 39). In CRC, Weifeng et al. reported associations between NOTCH1/2 expression and CD8+ T cell infiltration, though the underlying mechanisms remained undefined (40). Our work reveals that NOTCH3 alterations (both mutational and expression changes) regulate CD8+ T cell activation in CRC through the NOTCH3-RBPJ-PVR axis. Unlike the pleiotropic NOTCH3, which participates in diverse developmental and homeostatic processes, RBPJ’s more restricted function as a dedicated transcription factor may offer superior therapeutic tractability.

Our analysis revealed that NOTCH3 genetic mutations occur in approximately 7% of CRC cases - a frequency exceeding that of MSI-H tumors (41). In addition, the samples involved in the validation dataset (MSKCC-CRC and our dataset) are also mostly MSI samples, making it less convincing for the its predictive value in MSS samples. While NOTCH3-mutated tumors exhibit elevated MSI scores, these alterations may also hold clinical relevance for microsatellite-stable (MSS) patients. Furthermore, NOTCH3 expression levels demonstrated prognostic value regardless of immunotherapy status. This suggests that combined assessment of NOTCH3 mutation status and expression levels may enhance clinical stratification. The NOTCH3 mutant CRC samples exhibit significantly higher MSI score compared to the wild type, which suggest that NOTCH3 mutant may be influenced by, at least partially, by MSI status. In consistent with this, the alterations of NOTCH3 (both expression and mutation) were significantly associated with immune infiltration and immune activation related pathways, according to our result and previous studies (42), as a classical phenotype of MSI samples. Interestingly, while bulk transcriptomic analysis indicated an enrichment of M1 macrophages in NOTCH3-mutant tumors, scRNA-seq revealed a lower overall macrophage proportion in NOTCH3-high samples. This apparent discrepancy may reflect the technical differences between the methods and, more importantly, suggests that NOTCH3 alterations may not simply recruit more macrophages but rather promote a shift in their polarization towards a pro-inflammatory M1 phenotype, thereby shaping a more anti-tumor immune microenvironment.

We observed that the NOTCH3 genetic alteration rate was higher in uterine corpus endometrial carcinoma, ovarian serous cystadenocarcinoma, cutaneous melanoma, esophageal adenocarcinoma, adrenocortical carcinoma, gastric adenocarcinoma, and CRC. Notably, NOTCH3 mutations were significantly associated with activated immune-related pathways across cancers, including TCR, BCR, and TLR signaling. These pathways are crucial for immune activation and immunotherapy (43–46). We hypothesize cancers may share frequent genomic instability that could simultaneously promote NOTCH3 mutations and neoantigen production, thereby activating TCR/BCR pathways. Conversely, NOTCH3 alterations and their predictive value for immunotherapy may also benefit these cancer types. This requires further investigation. Although a high proportion of cancers showed significantly worse survival in NOTCH3-high samples, thymoma exhibited the opposite trend. The thymus is the central site of T-cell development, where NOTCH3 primarily functions by mediating T-cell differentiation. This may explain the distinct survival patterns observed in thymomas (tumors of the thymus) with NOTCH3 alterations.

While this study provides compelling evidence for the role of the NOTCH3-PVR axis in mediating immune evasion and immunotherapy resistance in colorectal cancer (CRC), several important limitations should be acknowledged. First, the clinical findings are derived from retrospective analyses rather than prospective validation cohorts, which may introduce selection bias and limit the reliability of NOTCH3 as a predictive biomarker. Second, the *in vivo* experiments were conducted in conventional immune proficient mouse models rather than humanized mice with reconstituted human immune systems, potentially compromising translational relevance given the known species-specific differences in NOTCH signaling and immune checkpoint interactions. Third, while we demonstrated that the R1669H mutation exhibits loss-of-function characteristics, the functional consequences of other NOTCH3 mutations remain uncharacterized, leaving open the possibility that some variants may retain partial or even gain-of-function activity in certain contexts. Fourth, the apparent enrichment of NOTCH3 alterations in dMMR/MSI-H tumors raises concerns about its applicability to pMMR/MSS CRC patients, who constitute over 95% of cases and represent the population with the greatest unmet need for immunotherapy biomarkers. Finally, while we identified PVR as a key downstream effector of NOTCH3-mediated immune suppression, the mechanistic scope remains narrow—a more comprehensive investigation of other potential NOTCH3-regulated immune checkpoints could reveal additional therapeutic targets and provide deeper insights into the network of immune evasion mechanisms orchestrated by NOTCH3 in CRC. Our *in vivo* efficacy experiments were performed using immunocompetent C57BL/6 mice implanted with human HCT116 cells. This model, while valuable for assessing therapy response within a functional mouse immune system, creates a species mismatch between human tumor-expressed PVR and the mouse TIGIT receptor on immune cells. Although studies have reported cross-reactivity between human PVR and mouse TIGIT (47) and the antibody targets mouse notch3, which lends biological plausibility to our observations, the binding affinity and downstream signaling may not fully recapitulate the human interaction. Therefore, the specific contribution of the PVR-TIGIT axis to the enhanced efficacy of anti-PD-L1 therapy upon NOTCH3 inhibition should be interpreted with this caveat. Future studies using humanized mouse models (48) would be valuable to confirm these findings in a fully human PVR-TIGIT context. Addressing these limitations through prospective clinical studies, humanized animal models, systematic functional characterization of NOTCH3 variants, and expanded mechanistic exploration will be critical for validating the clinical utility of NOTCH3 as a predictive biomarker and therapeutic target, particularly in the pMMR/MSS setting where effective immunotherapy strategies are urgently needed.

## Data Availability

The original contributions presented in the study are included in the article/**Supplementary Material**. Further inquiries can be directed to the corresponding author/s.
