# Supplementary figures and images for: NOTCH3 attenuates cytotoxicity via RBPJ-dependent PVR upregulation to influence immunotherapy outcomes in colorectal cancer
Source: Front Immunol. 2026 Feb 23;17:1741261. doi: 10.3389/fimmu.2026.1741261 (PMC12967962; doi:10.3389/fimmu.2026.1741261)

## Overall survival

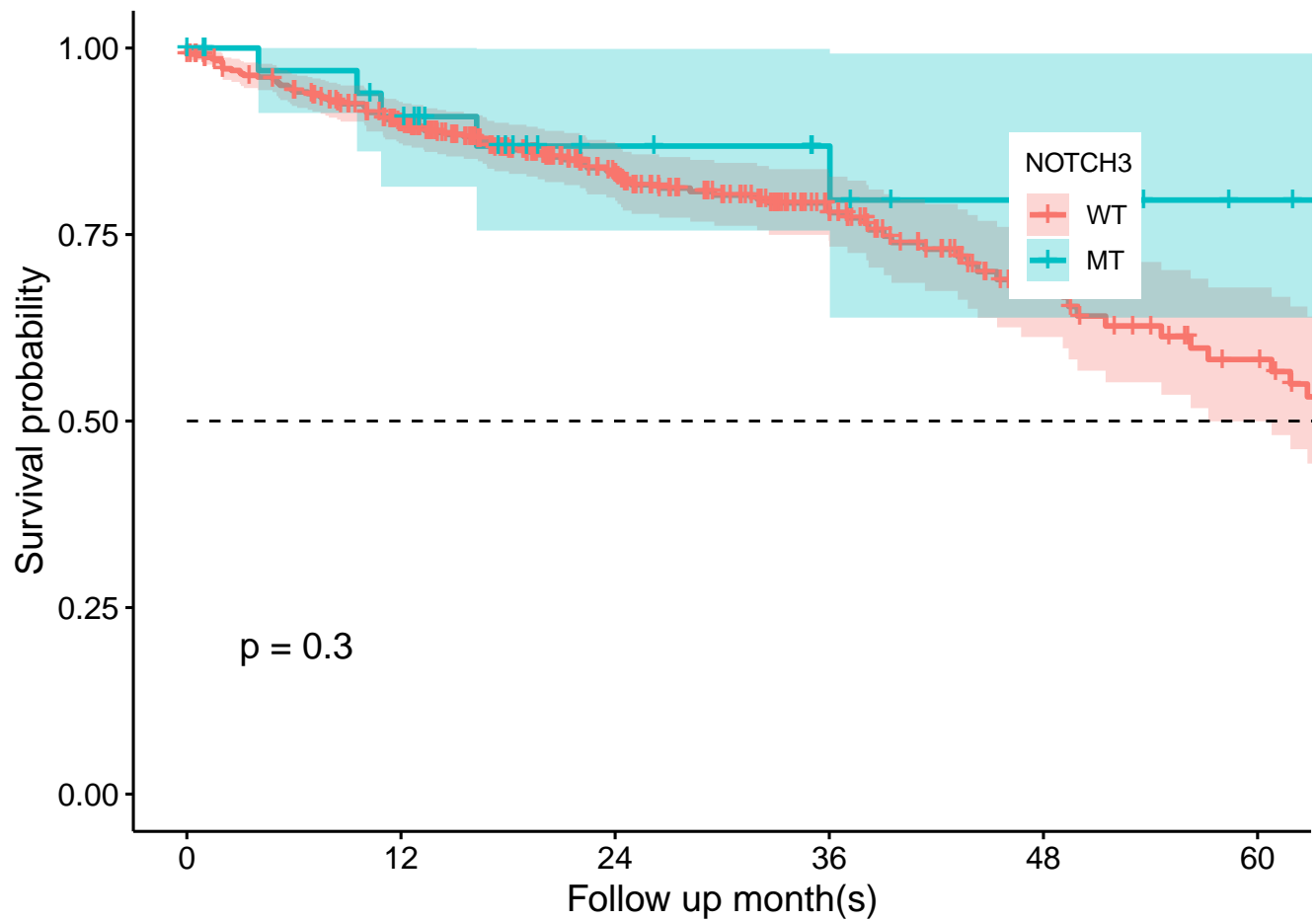

## Number at risk

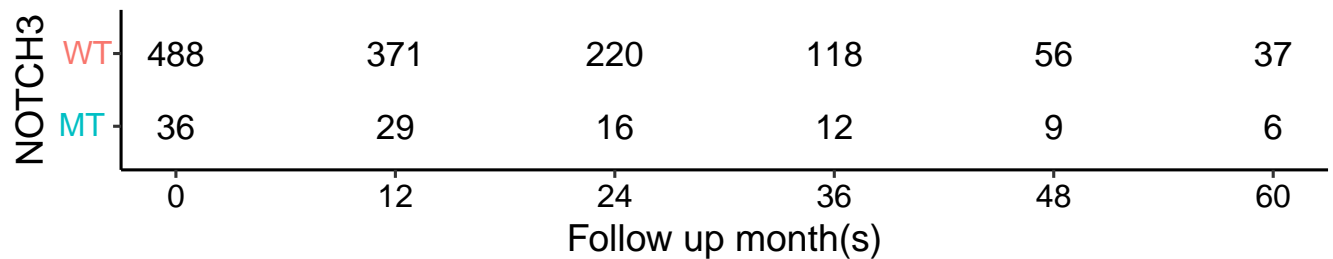

Supplement: Supplementary file 1 [file DataSheet1.pdf]

XCELL

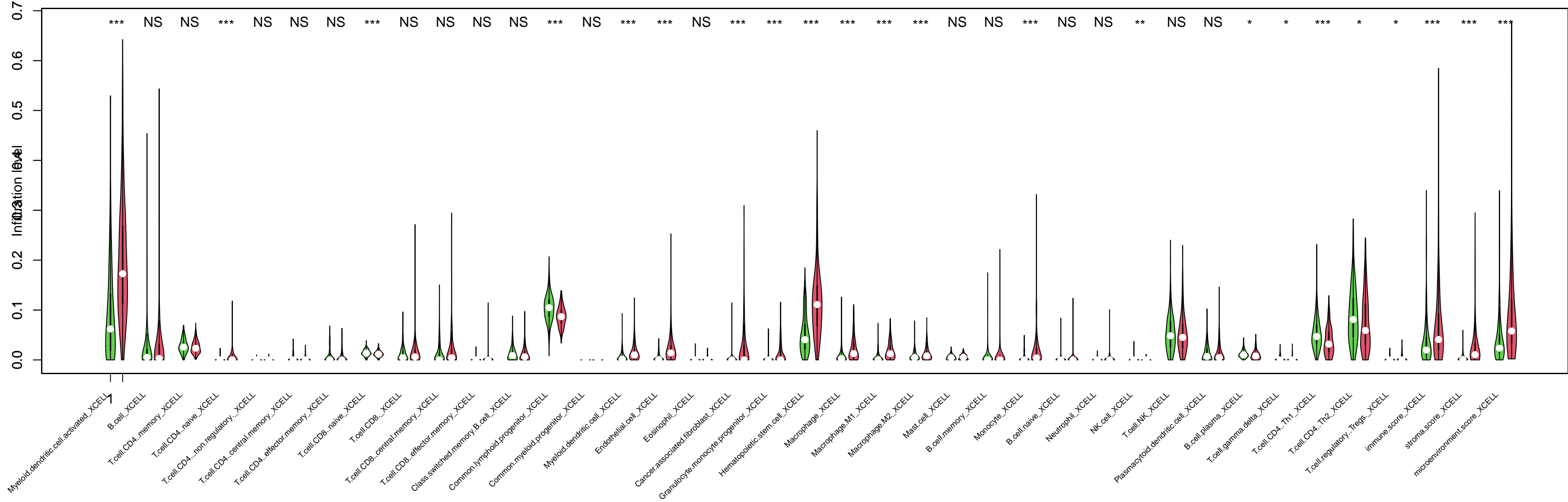

# CIBERSORT

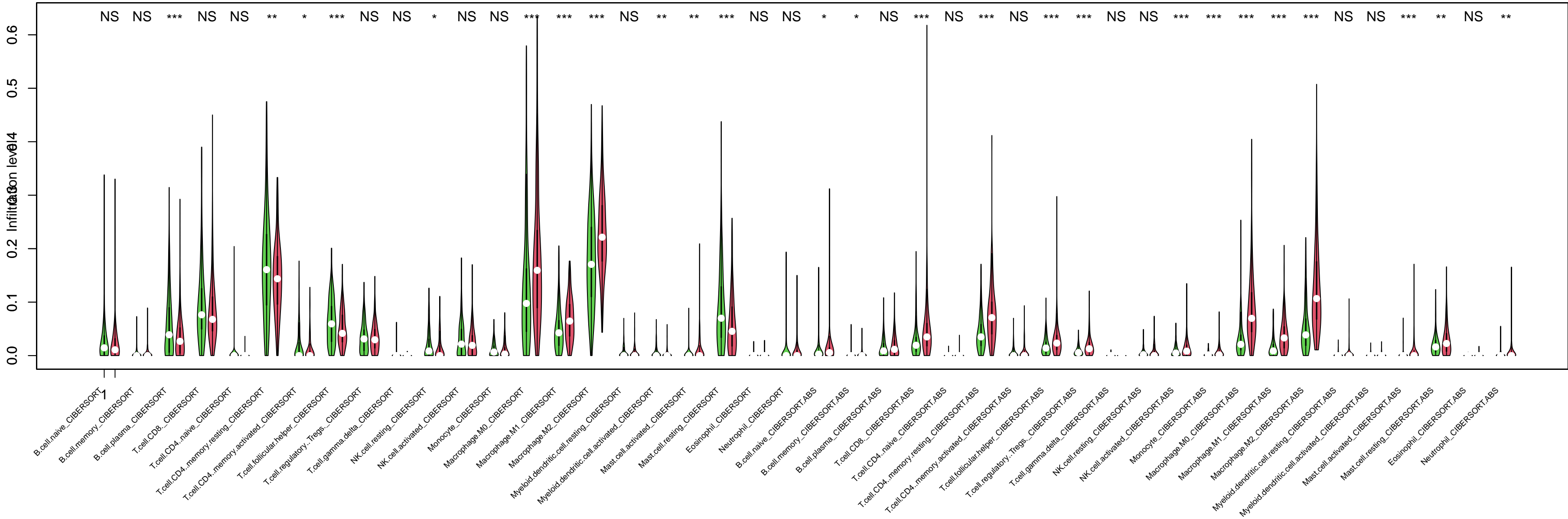

# Other

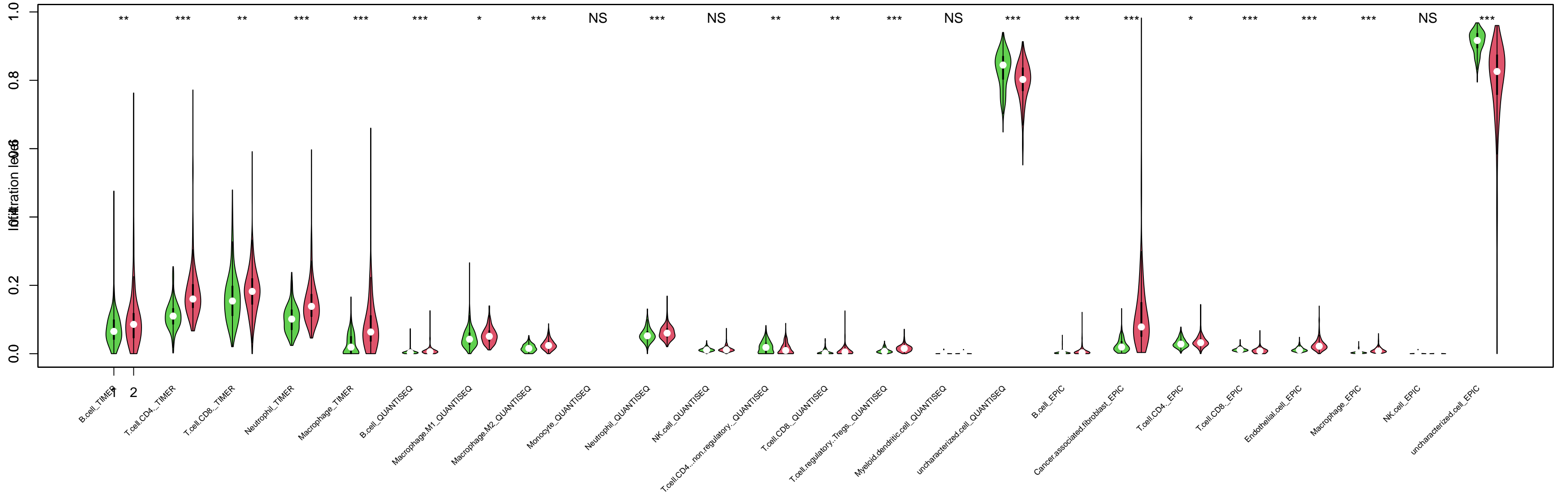

Supplement: Supplementary file 2 [file DataSheet2.pdf]

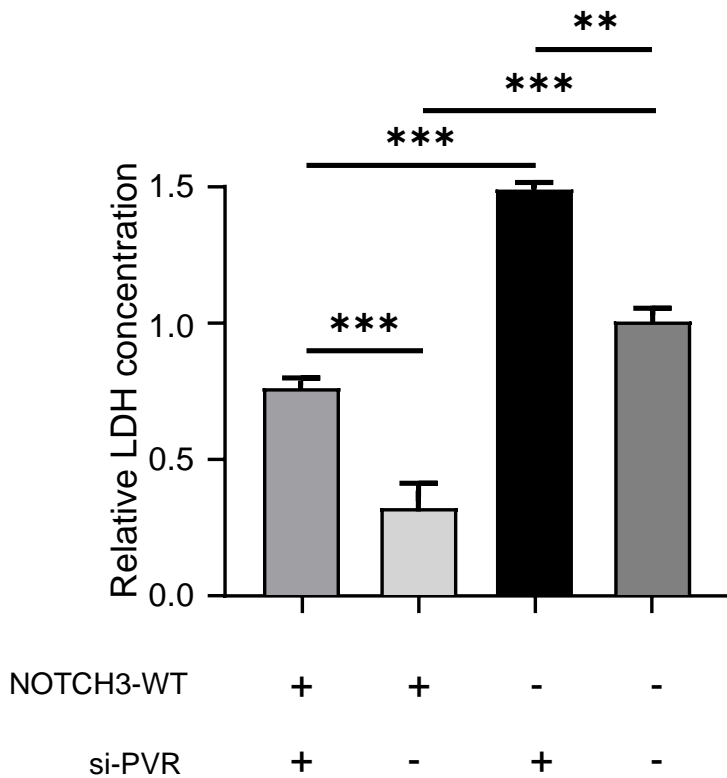

Supplement: Supplementary file 3 [file DataSheet3.pdf]

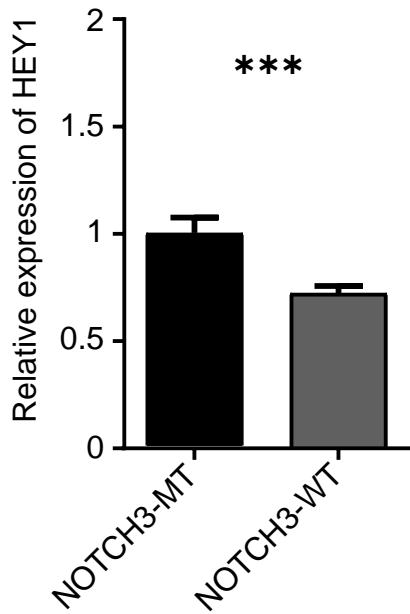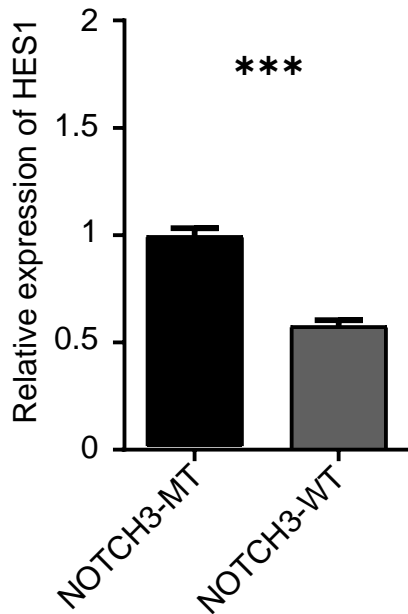

Supplement: Supplementary file 4 [file DataSheet4.pdf]

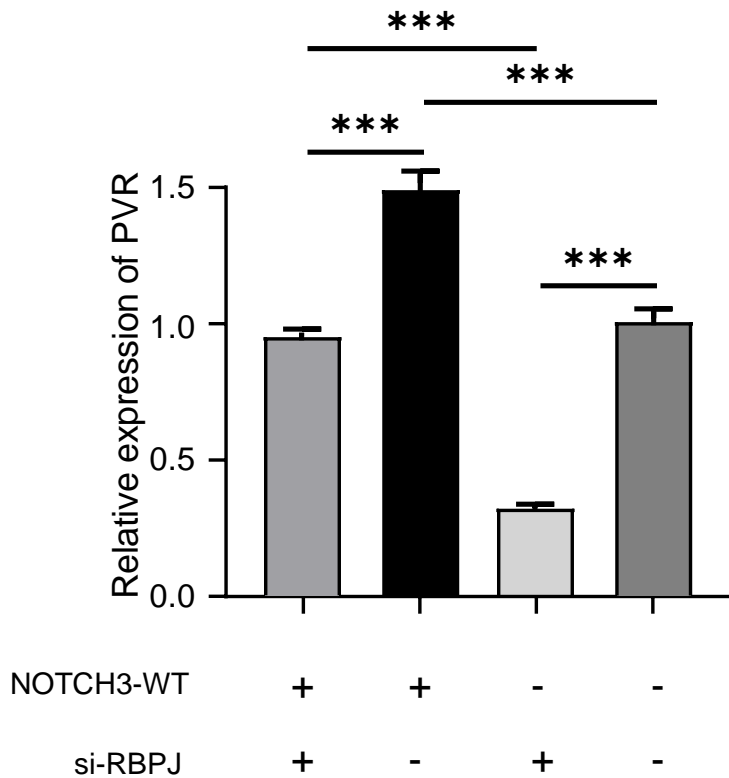

Supplement: Supplementary file 5 [file DataSheet5.pdf]

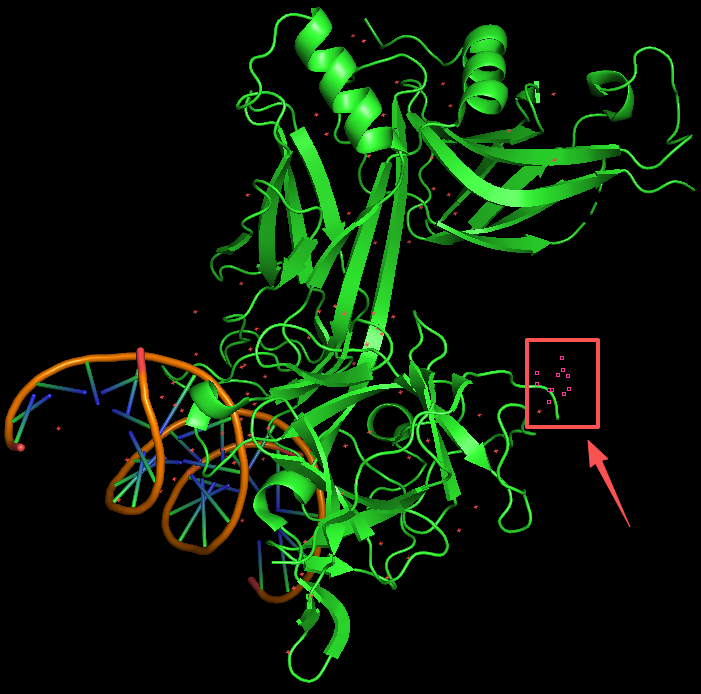

Supplement: Supplementary file 6 [file Image1.png]
